# A Complex Relationship between Visfatin and Resistin and microRNA: An In Vitro Study on Human Chondrocyte Cultures

**DOI:** 10.3390/ijms19123909

**Published:** 2018-12-06

**Authors:** Sara Cheleschi, Nicola Giordano, Nila Volpi, Sara Tenti, Ines Gallo, Martina Di Meglio, Stefano Giannotti, Antonella Fioravanti

**Affiliations:** 1 Rheumatology Unit; Department of Medicine, Surgery and Neuroscience, Azienda Ospedaliera Universitaria Senese, Policlinico Le Scotte, 53100 Siena, Italy; saracheleschi@hotmail.com (S.C.); sara_tenti@hotmail.it (S.T.); ins.gll3@gmail.com (I.G.); 2 Scleroderma Unit, Department of Medicine, Surgery and Neuroscience, University of Siena, Policlinico Le Scotte, 53100 Siena, Italy; nicola.giordano@unisi.it; 3 Neurology Unit, Department of Medicine, Surgery and Neuroscience, Azienda Ospedaliera Universitaria senese, Policlinico Le Scotte, 53100 Siena, Italy; nila.volpi@unisi.it; 4 Department of Medicine, Surgery and Neurosciences, Section of Orthopedics and Traumatology, University of Siena, Policlinico Le Scotte, 53100 Siena, Italy; martina.dimeglio@gmail.com (M.D.M.); stefano.giannotti@unisi.it (S.G.)

**Keywords:** visfatin, resistin, adipokines, osteoarthritis, miRNA, chondrocyte, T/C-28a2, NF-κB

## Abstract

Growing evidence indicates the important role of adipokines and microRNA (miRNA) in osteoarthritis (OA) pathogenesis. The purpose of the present study was to investigate the effect of visfatin and resistin on some miRNA (34a, 140, 146a, 155, 181a, let-7e), metalloproteinases (MMPs), and collagen type II alpha 1 chain (Col2a1) in human OA chondrocytes and in the T/C-28a2 cell line. The implication of nuclear factor (*NF)-κB* in response to adipokines was also assessed. Chondrocytes were stimulated with visfatin (5 or 10 μg/mL) and resistin (50 or 100 ng/mL) with or without NF-κB inhibitor (BAY-11-7082, 1 μM) for 24 h. Viability and apoptosis were detected by MMT and cytometry, miRNA, MMP-1, MMP-13, and Col2a1 by qRT-PCR and *NF-κB* activation by immunofluorescence. Visfatin and resistin significantly reduced viability, induced apoptosis, increased *miR-34a*, *miR-155*, *miR-181a*, and *miR-let7e*, and reduced *miR-140* and *miR-146a* gene expression in OA chondrocytes. *MMP-1*, *MMP-13*, and *Col2a1* were significantly modulated by treatment of OA chondrocytes with adipokines. Visfatin and resistin significantly increased NF-κB activation, while the co-treatment with BAY11-7082 did not change *MMPs* or *Col2a1* levels beyond that caused by single treatment. Visfatin and resistin regulate the expression levels of some miRNA involved in OA pathogenesis and exert catabolic functions in chondrocytes via the *NF-κB* pathway. These data confirm the complex relationship between adipokines and miRNA.

## 1. Introduction

Osteoarthritis (OA) represents the most widespread chronic degenerative joint disorder and is a leading cause of chronic disability, impairment, and reduced quality of life in adult and elderly populations [1]. Osteoarthritis is generally accepted to be caused by physical and chemical degenerative changes of the joint tissue, resulting in a progressive degeneration of articular cartilage, osteophyte formation, and synovial membrane low-grade inflammation, leading to the loss of function and pain [2,3,4,5].

Obesity constitutes one of the most important risk factors for OA in non-weight and weight bearing joints [6,7,8,9].

Growing evidence indicates that white adipose tissue is an active endocrine organ producing multiple factors, known as adipocytokines, such as interleukin (IL)-6, tumor necrosis factor (TNF)-α, as well as adipokines: adiponectin, leptin, resistin, chemerin, and visfatin [10]. Adipokines have shown pleiotropic effects modulating the immune response and affecting bone and cartilage metabolisms [10,11].

Recently, resistin has received more attention for its involvement in cartilage damage. In human OA chondrocyte cultures, resistin induces the expression of different pro-inflammatory cytokines and chemokines, as well as of metalloproteinases (MMP)-1, MMP-13, and a disintegrin and metalloproteinase with thrombospondin motifs (ADAMTS)-4 [12]. Serum levels of resistin are increased in patients with OA of the hand [13,14] and in serum and synovial fluid of patients with knee OA [15,16,17].

Visfatin has pro-inflammatory, immunomodulating, and pro-degradative effects on cartilage [18,19,20]. Serum visfatin levels are increased in patients with knee or hand OA [14,21,22].

MicroRNA (miRNA) are 22–25 nucleotides and non-coding, single-stranded RNA molecules which act in post-transcriptional regulation of gene expression either through enhancement or degradation of the target gene messenger RNA (mRNA) [23]. Different miRNA expression profiles were observed in OA cartilage with respect to normal samples [24,25,26,27,28,29]. Some miRNA dysregulated in OA target genes encoding for proteins implicated in extracellular matrix remodeling and in pro-inflammatory activities [30,31].

A mutual interaction between miRNA and adipokines has been recently reported; miRNA modulate adipocyte differentiation in mouse and human cell line models, and some adipokines are involved in the regulation of miRNA expression [32,33].

On the basis of these considerations we hypothesized the possible involvement of adipokines in the regulation of some miRNA implicated in OA pathogenesis.

For this purpose, we investigated the possible effect of visfatin and resistin in the viability and apoptosis, as well as in the regulation of the expression levels of a pattern of miRNA (*miR-34a*, *miR-146a*, *miR-155*, *miR-181a*, *miR-140*, and *miR-let7e*) in human OA chondrocyte cultures and in T/C-28a2 cell lines. The modifications of the main extracellular matrix-degrading enzymes, *MMP-1*, *MMP-13*, and of collagen type II alpha 1 chain (*Col2a1*) were also analyzed. Moreover, the possible involvement of the nuclear factor (*NF)-κB* signaling pathway was investigated by means of immunofluorescence (IF) and semi-quantitative analysis, and by using a specific *NF-κB* inhibitor.

## 2. Results

### 2.1. Cell Viability Assay

**Figure 1:**
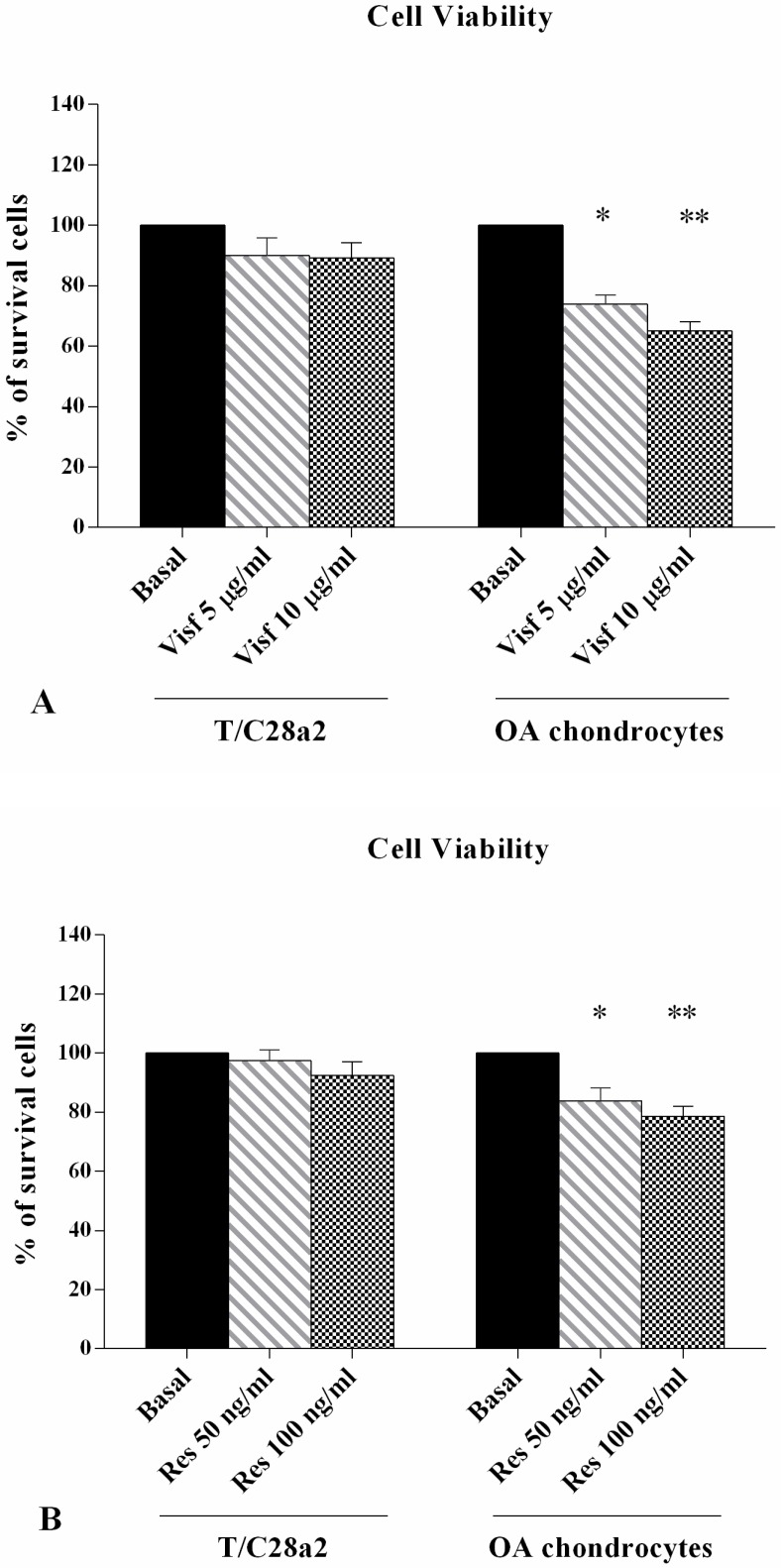
Evaluation of cell viability by MTT assay in the T/C-28a2 cell line and in human OA chondrocytes. Cells were evaluated at basal conditions and after 24 h of stimulus with visfatin (5 μg/mL and 10 μg/mL) (**A**) and resistin (50 ng/mL and 100 ng/mL) (**B**). Data were expressed as percentage of cell viability in all the studied conditions. The percentage was referenced to the ratio of the value of interest and basal conditions. The value of basal conditions was reported equal to 100. Data were expressed as mean ± SD of triplicate values. * *p* < 0.05, ** *p* < 0.01 versus basal conditions. Visf = Visfatin, Res = Resistin.

Cell viability evaluated by MTT test is reported in Figure 1. A significant decrease in the percentage of survival cells was observed after stimulus of OA chondrocytes with visfatin 5 μg/mL and 10 μg/mL (*p* < 0.05, *p* < 0.01, respectively, Figure 1A) and resistin 50 ng/mL and 100 ng/mL (*p* < 0.05, *p* < 0.01, respectively, Figure 1B), in comparison to basal conditions in a dose-dependent manner. No modification in the T/C-28a2 cell line was observed. The data was confirmed by Trypan Blue test.

### 2.2. Apoptosis Detection

**Figure 2:**
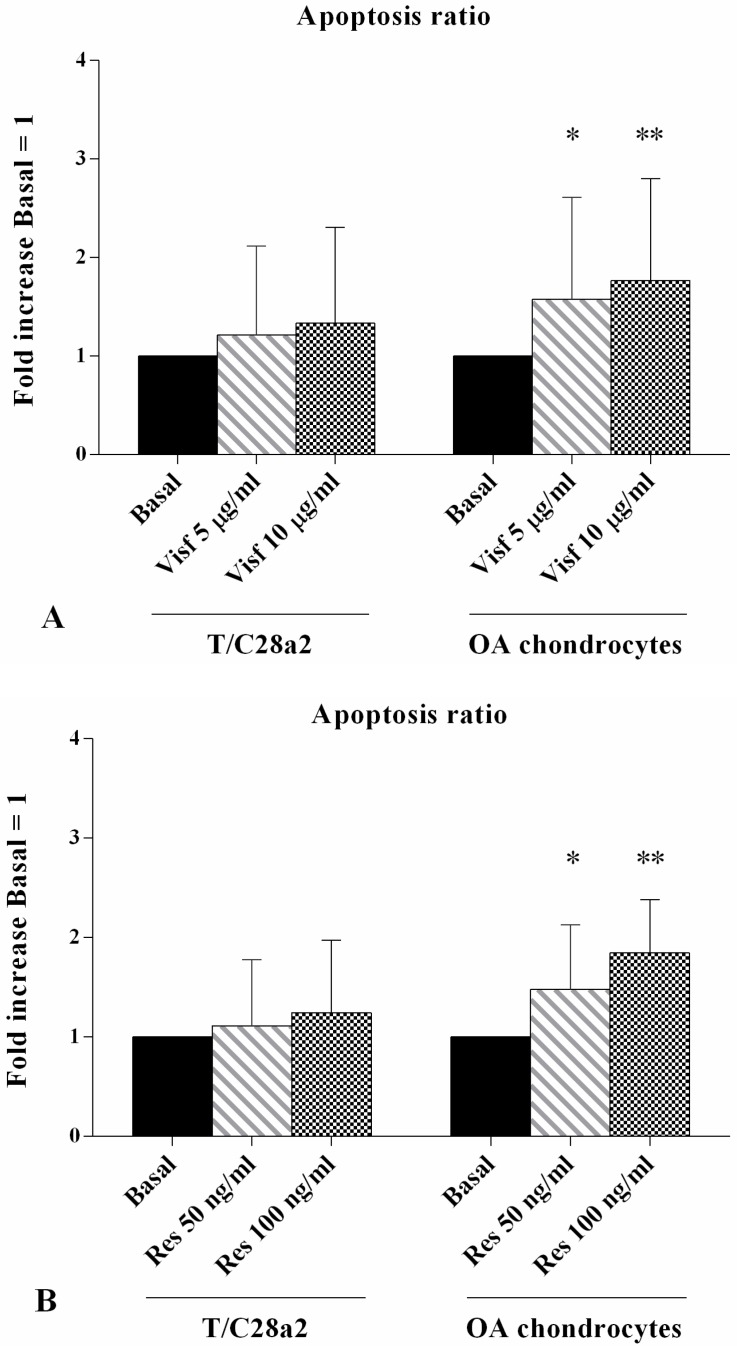
Apoptosis detection by flow cytometry in T/C-28a2 cell line and in human osteoarthritis (OA) chondrocytes. Cells were evaluated at basal conditions and after 24 h of stimulus with visfatin (5 μg/mL and 10 μg/mL) (**A**) and resistin (50 ng/mL and 100 ng/mL) (**B**). Apoptosis was measured with Annexin Alexa fluor 488 assay. Data were expressed as percentage of positive cells for Annexin-V and propidium iodide (PI) in all the studied conditions. The ratio of apoptosis was referenced to the ratio of the value of interest and basal conditions. The value of basal conditions was reported equal to 1. Data were expressed as mean ± SD of triplicate values. * *p* < 0.05, ** *p* < 0.01 versus basal conditions. Visf = Visfatin, Res = Resistin.

Figure 2 summarizes the results on the ratio of chondrocyte apoptosis obtained by flow cytometry analysis. The stimulus of OA cells with visfatin 5 μg/mL and 10 μg/mL induced a significant increase of apoptotic chondrocytes (*p* < 0.05, *p* < 0.01, respectively, Figure 2A) in comparison to the basal time. Resistin at a concentration of 50 ng/mL and 100 ng/mL also determined a statistically increase of the apoptosis ratio (*p* < 0.05, *p* < 0.01, respectively, Figure 2B). No detectable changes were observed in T/C-28a2 cell line.

### 2.3. Gene Expression of miRNA

**Figure 3:**
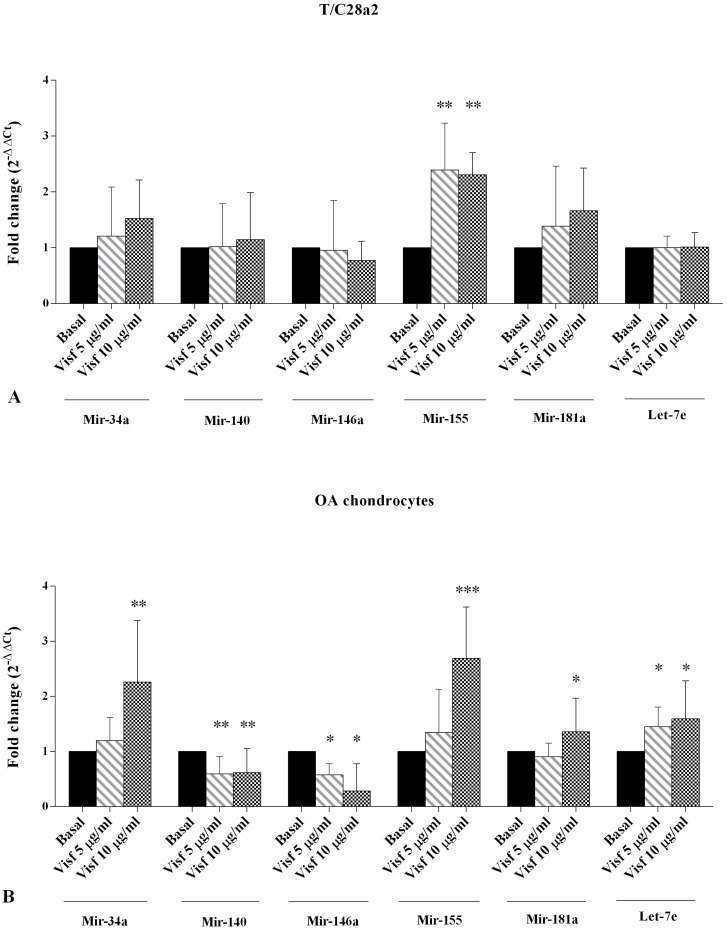

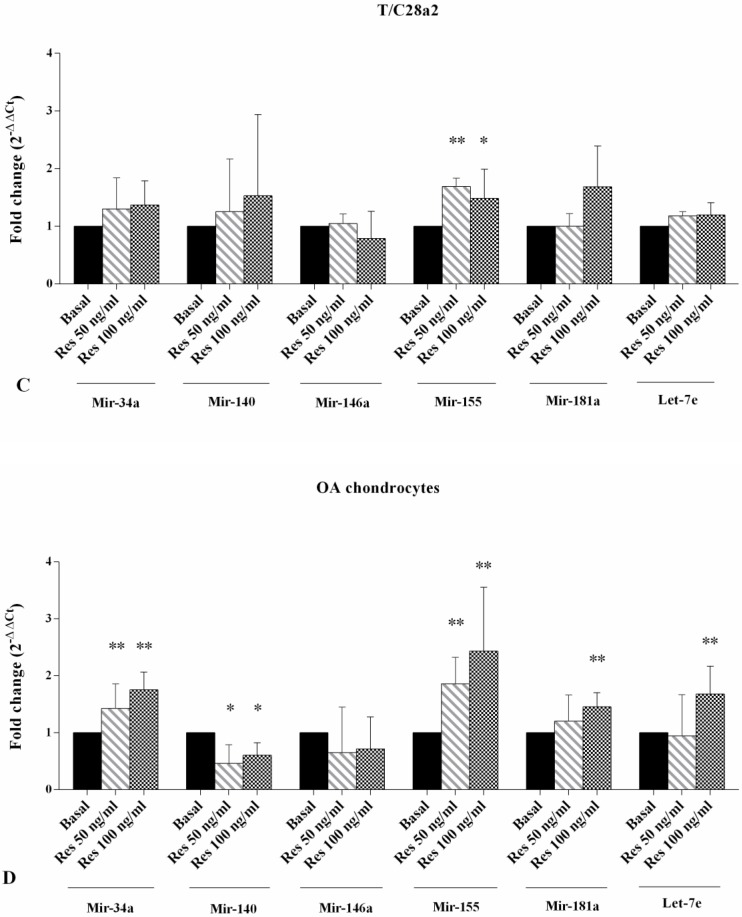
Expression levels of *miR-34a*, *miR-140*, *miR-155*, *miR-181a*, and *miR-let7e* by real-time PCR in the T/C-28a2 cell line (**A**,**C**) and in human OA chondrocytes (**B**,**D**). Cells were evaluated at basal conditions and after 24 h of stimulus with visfatin (5 μg/mL and 10 μg/mL) and resistin (50 ng/mL and 100 ng/mL). The gene expression was referenced to the ratio of the value of interest and basal conditions. The value of basal conditions was reported equal to 1. Data were expressed as mean ± SD of triplicate values. * *p* < 0.05, ** *p* < 0.01, *** *p* < 0.001 versus basal conditions. Visf = Visfatin, Res = Resistin.

Visfatin 5 μg/mL and 10 μg/mL (*p* < 0.01) induced a significant increase of *miR-155* expression levels in the T/C-28a2 cell line, while it did not influence *miR-34a*, *miR-140*, *miR-146a*, *miR-181a*, and *miR-let7e* expressions (Figure 3A). In OA chondrocytes (Figure 3B), visfatin, 5 μg/mL and 10 μg/mL, was able to significantly decrease *miR-140* and *miR-146a* (*p* < 0.01, *p* < 0.05, respectively) and significantly increase *miR-let7e* (*p* < 0.05) expression levels when compared with basal conditions. *MiR-34a*, *miR-155*, and *miR-181a* were significantly increased by the higher concentration of visfatin (*p* < 0.01, *p* < 0.001, *p* < 0.05, respectively).

Resistin 50 ng/mL and 100 ng/mL significantly increased the expression levels of *miR-155* (*p* < 0.01, *p* < 0.05, respectively) in the T/C-28a2 cell line in comparison to basal state (Figure 3C). Moreover, resistin at both studied concentrations induced a significant upregulation of *miR-34a* (*p* < 0.01), *miR-155* (*p* < 0.01), *miR-181a* (*p* < 0.01 for resistin 100 ng/mL), and *miR-let7e* (*p* < 0.01 for resistin 100 ng/mL), and a downregulation of *miR-140* (*p* < 0.05) gene expression in OA chondrocytes (Figure 3D).

### 2.4. Gene Expression of MMP-1, MMP-13, and Col2a1

**Figure 4:**
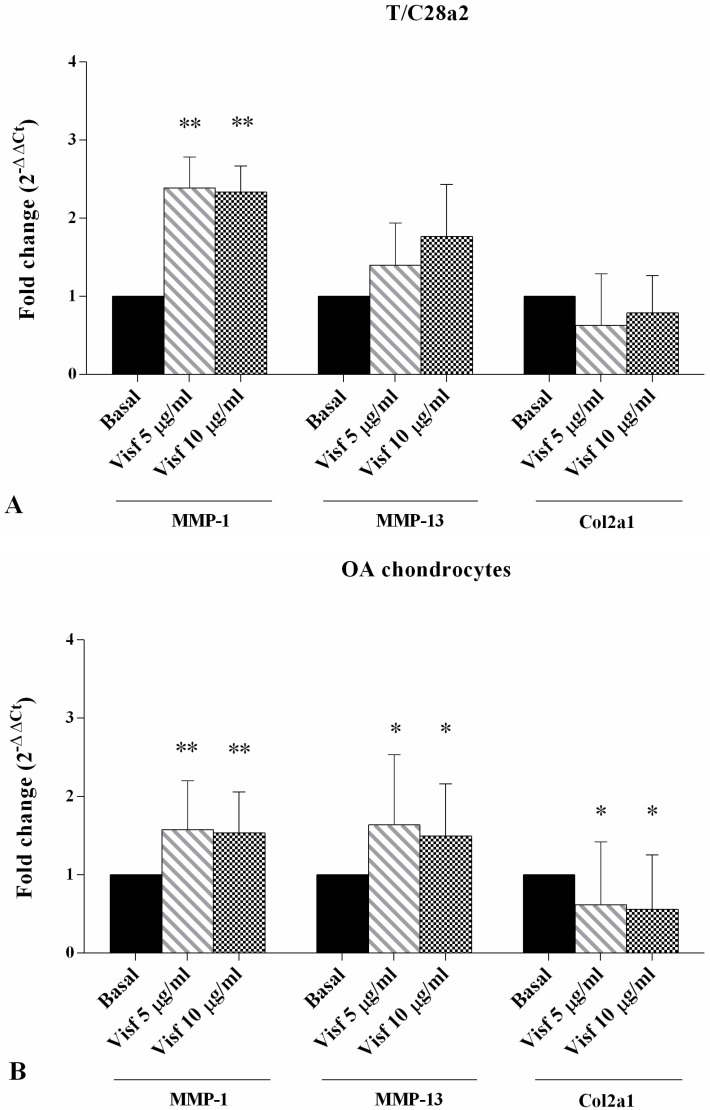

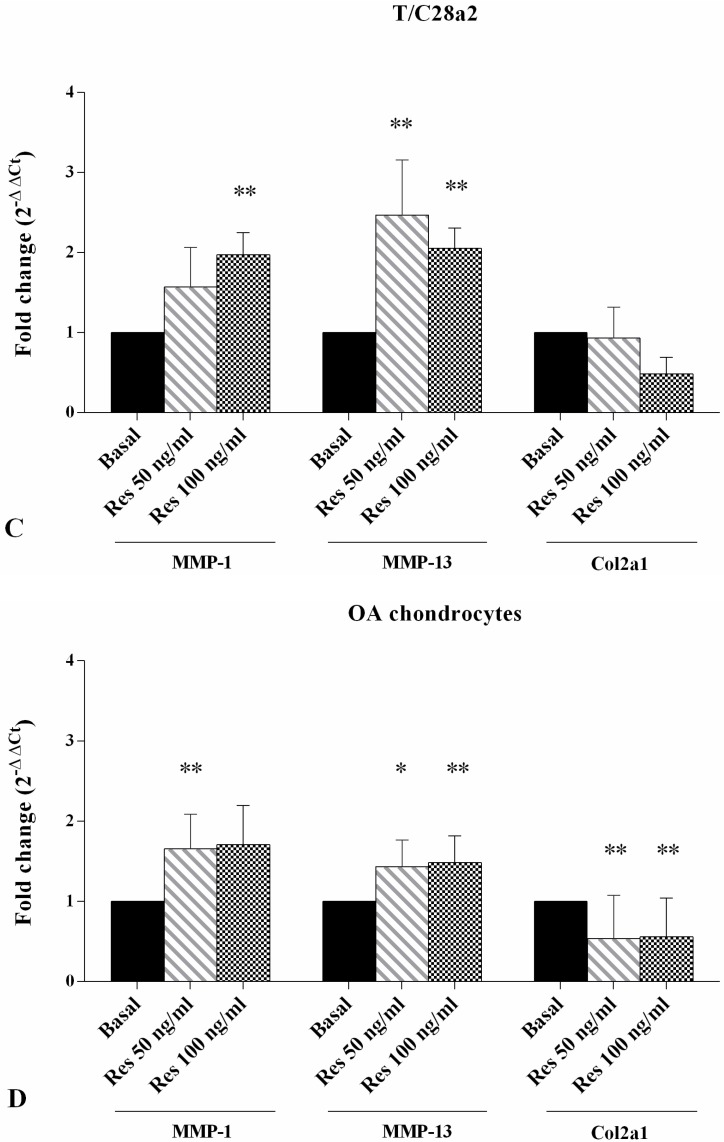
Expression levels of *MMP-1*, *MMP-13*, and *Col2a1* by real-time PCR in the T/C-28a2 cell line (**A**,**C**) and in human OA chondrocytes (**B**,**D**). Cells were evaluated at basal conditions and after 24 h of stimulus with visfatin (5 μg/mL and 10 μg/mL) and resistin (50 ng/mL and 100 ng/mL). The gene expression was referenced to the ratio of the value of interest and basal conditions. The value of basal conditions was reported equal to 1. Data were expressed as mean ± SD of triplicate values. * *p* < 0.05, ** *p* < 0.01 versus basal conditions. Visf = Visfatin, Res = Resistin.

In T/C-28a2 cell line, a significant increase of *MMP-1* gene expression was observed after stimulus with visfatin 5 μg/mL and 10 μg/mL (*p* < 0.01) in comparison to basal conditions. No changes were observed in *MMP-13* and *Col2a1* (Figure 4A). The incubation of OA chondrocytes with visfatin 5 μg/mL and 10 μg/mL determined a significant upregulation of *MMP-1* (*p* < 0.01), *MMP-13* (*p* < 0.05), and a downregulation of *Col2a1* (*p* < 0.05) expression levels (Figure 4B).

Resistin 50 ng/mL significantly increased the gene expression of *MMP-13* (*p* < 0.01) in the T/C-28a2 cell line and upregulated the transcriptional levels of *MMP-1* and *MMP-13* when tested at concentration of 100 ng/mL (*p* < 0.01), in comparison to basal time (Figure 4C). In OA cells, the incubation with resistin 50 ng/mL and 100 ng/mL significantly increased the expression levels of *MMP-1* (*p* < 0.01 for resistin 50 ng/mL) and *MMP-13* (*p* < 0.05, *p* < 0.01, respectively) and decreased *Col2a1* (*p* < 0.01) (Figure 4D).

### 2.5. Immunofluorescence Analysis

**Figure 5:**
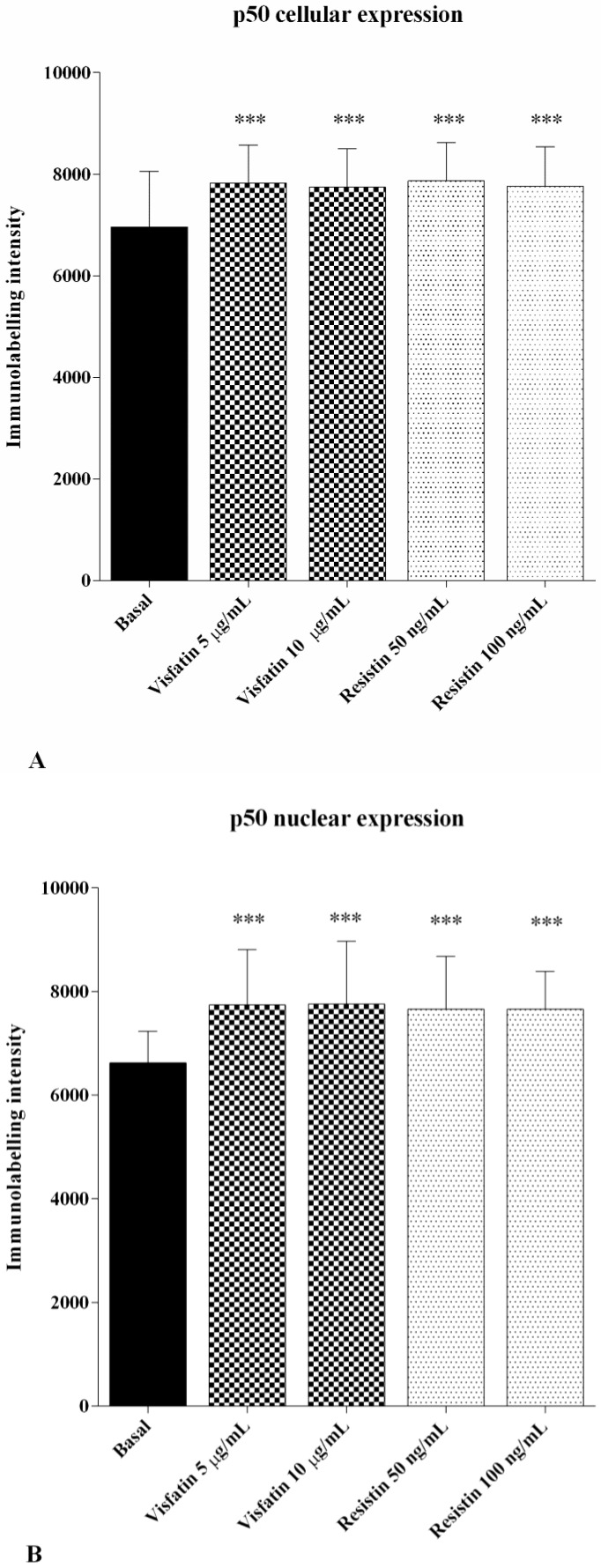
Immunofluorescence labelling of p50 *NF-κB* subunit localization of human OA chondrocytes. Cells were evaluated at basal conditions and after 24 h of stimulus with visfatin (5 μg/mL and 10 μg/mL) and resistin (50 ng/mL and 100 ng/mL). In chondrocytes stimulated with adipokines, p50 cytoplasmic upregulation and p50 nuclear translocation were observed (**A**,**B**) The histograms of immunolabelling intensity were plotted for the nuclear and cytoplasm expression for p50 subunit. *** *p* < 0.001 versus basal conditions.

Figure 5A,B reported the activation and the nuclear translocation of the *NF-κB* subunit in OA chondrocytes. The signal of p50 *NF-κB* was consistently detected in the cytoplasm and in the nucleus at basal conditions. Visfatin and resistin significantly increased p50 cytoplasmic synthesis (*p* < 0.001, Figure 5A), with a resulting higher immunolabelling intensity, as well as its activation, as indicated by nuclear translocation (*p* < 0.001, Figure 5B), in comparison to baseline.

### 2.6. Regulation of Gene Expression of MMP-1, MMP-13 and Col2a1 after NF-κB Inhibition

**Figure 6:**
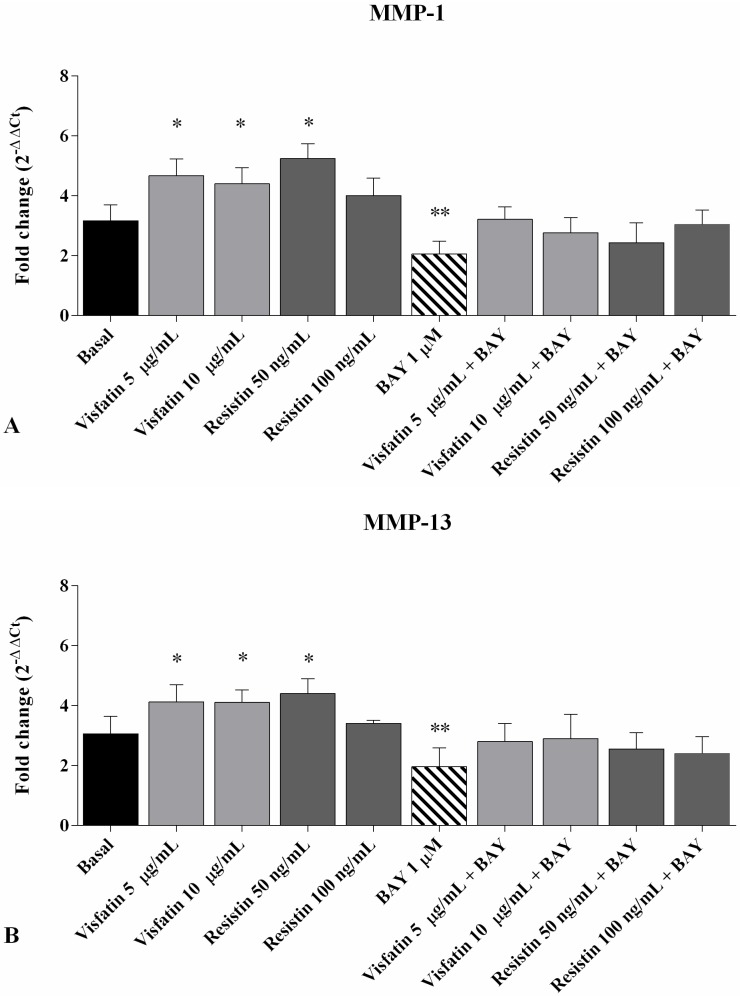

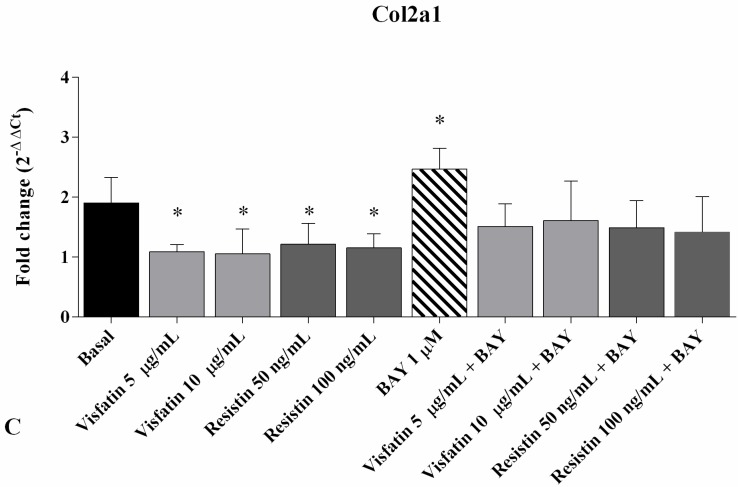
Expression levels of *MMP-1* (**A**), *MMP-13* (**B**), and *Col2a1* (**C**) by real-time PCR in human OA chondrocytes. Cells were evaluated at basal conditions, pre-incubated for 2 h with 1 μM BAY 11-7082 (*NF-κB* inhibitor) and after 24 h of stimulus with visfatin (5 μg/mL and 10 μg/mL) and resistin (50 ng/mL and 100 ng/mL). Data were expressed as mean ± SD of triplicate values. * *p* < 0.05, ** *p* < 0.01 versus basal conditions.

In Figure 6 we showed that the incubation of OA chondrocytes with visfatin (5 μg/mL and 10 μg/mL) and resistin (50 ng/mL and 100 ng/mL) significantly upregulated *MMP-1* (*p* < 0.05 for visfatin 5 μg/mL and 10 μg/mL, and resistin 50 ng/mL, Figure 6A), *MMP-13* (*p* < 0.05 for visfatin 5 μg/mL and 10 μg/mL, and resistin 50 ng/mL, Figure 6B) and downregulated *Col2a1* (*p* < 0.05, Figure 6C) expression levels in comparison to basal conditions.

The pre-incubation of OA chondrocytes with a well-known *NF-κB* inhibitor, BAY 11-7082 (IKKα/β), reduced *MMP-1* (*p* < 0.01), *MMP-13* (*p* < 0.01), and increased *Col2a1* (*p* < 0.05) gene expression, in a significant manner, with respect to the basal state.

Chondrocytes co-treated with visfatin, resistin plus BAY11-7082 did not exhibit enhanced effects in *MMP-1*, *MMP-13* and *Col2a1* expression levels compared to cells treated with visfatin, resistin or BAY11-7082 alone.

## 3. Discussion

Obesity is considered one of the most important risk factors for OA incidence, progression, and disability [9]. The mechanism by which obesity influences the structural damage of cartilage is not fully elucidated. A potential role was assigned to adipokines [10,34,35]; however, their exact effect on cartilage metabolism have not been completely established and controversial results have been found in chondrocytes [12,36,37].

Along with the well-known role of miRNA in the development and in the progression of OA [24], recent evidence has pointed out the involvement of these factors also in the regulation of adipogenesis, obesity, insulin resistance, and diabetes [38].

According to previous studies, we tested the effects of two different concentrations of visfatin and resistin in chondrocyte cultures focusing on apoptosis, on different miRNA, and on the main factors involved in cartilage metabolism [12,39].

Apoptosis represents a regulated active mechanism of cell death involved in development, homeostasis, and the aging processes, such as OA [40]. Our results demonstrated the pro-apoptotic effect of visfatin and resistin in OA chondrocytes. Gosset et al. [39] did not observe any significant modification in cell viability of OA cells treated with visfatin for 6 h; the discrepancy with our data can be attributed to the shorter timing of visfatin treatment. A more recent study, performed in visfatin-stimulated endothelial cells for 48 h, demonstrated a significant increase of apoptosis and the upregulation of two of the main proteins implicated in the regulation of this process, caspase-3 and Bax [41], supporting our results. Concerning resistin stimulus, to our knowledge, no studies have been performed on chondrocytes cultures; treatment of human breast cancer and myeloma cell lines with resistin reduced the apoptotic cell in a significant dose- and time-dependent manner, resulting in a growth of survival and resistance [42,43]. Our data appear in contrast with the updated literature; however, this could be explained by the experimental procedures differently employed, such as the cellular type analyzed, and the treatment conditions applied.

After the incubation of OA chondrocytes with visfatin and resistin, we demonstrated a significant modulation of a pattern of miRNA implicated in OA pathogenesis, and of some factors involved in cartilage metabolism.

Recent evidence reveals that the increased expression levels of *miR-34a* caused apoptosis and limited cell proliferation in human OA chondrocytes [44]. Our data reported a significant overexpression of *miR-34a* in visfatin and resistin stimulated cultures. These results are supported by the study of Wen et al. [45], demonstrating the upregulation of *miR-34a* after the stimulus of HepG2 cells with resistin for 24 h. At the moment, no previous data about the interaction between visfatin and miR-34a are available.

Furthermore, we can hypothesize that the observed negative effects on apoptosis and cell survival by these adipokines are mediated by miR-34a.

A reduced expression of *miR-140* has been reported in OA chondrocytes in comparison to normal cells, contributing to regulate the differentiation and proliferation of hypertrophic chondrocytes and the production of some inflammatory cytokine, as well as bone development and cartilage homeostasis [46,47,48]. Furthermore, some in vitro studies on chondrocytes observed the downregulation of *miR-140* induced by pro-inflammatory cytokines [46,47]. Similarly, the stimulus of our OA cultures by visfatin and resistin significantly reduced the expression levels of *miR-140*.

To our knowledge, no previous data are available and comparable to the results of the present paper; however, a study carried out on ATDC5 chondrocytes cell line demonstrated the regulation of *miR-140* after treatment with serum containing leptin [49].

Transfection experiments on animal models and on chondrocyte cultures identified MMP-13 and ADAMTS-5 as direct target genes of miR-140 [50,51].

Metallopreteinase-1 and MMP-13 contribute to promote cartilage breakdown by the degradation of proteoglycans and Col2a1, the main components of the articular extracellular matrix [52,53].

Previous data reported a significant production of *MMP-1* and *MMP-13*, and a decrease of *Col2a1* in OA chondrocytes in comparison to normal cells [12,29,53,54].

In this study we showed an increase of the gene expression of *MMP-1* and *MMP-13* and a downregulation of *Col2a1* mRNA levels in OA chondrocytes after 24 h of the stimulus with visfatin and resistin compared to OA non-stimulated cells, highlighting and confirming the involvement of these adipokines in cartilage degradation processes. These data are confirmed by several studies performed on chondrocytes cultures [12,15,39,55,56,57].

Lately, transfection experiments on human chondrocytes have been performed, showing the negative regulation of *miR-146a* on *MMP-13* and *ADAMTS-5* levels, indicating the anti-catabolic property of the miRNA [58].

MiRNA-146a is largely expressed in different species and tissues exerting a pivotal role in inflammatory and immune processes and participating in chondrocytes anabolic/catabolic balance [59]; however, data about the pattern of miR-146 expression in OA are controversial [48,60,61]. Some *in vitro* studies demonstrated an opposite modulation of this miRNA after different kinds of stimuli. Indeed, an increase in *miR-146a* gene expression after the stimulus of OA chondrocytes with IL-1β [61] or after cycles of mechanical injuring pressure [28] was observed, while reduced expression in hydrogen peroxide (H_2_O_2_)-stimulated chondrocytes was detected [48]. Our results showed, for the first time, a significant decrease in the expression levels of *miR-146a* in human OA chondrocytes treated with visfatin and a slight, but not significant, reduction after resistin incubation.

Mir-181a and miR-155 have been firstly related to the maturation and the activation of the immune system cells and to the regulation of inflammatory processes in autoimmune diseases and in arthritis [62,63]. These miRNA were found increased in the PBMC of OA patients and in human OA chondrocytes compared to normal cells [64,65,66]. It has been shown that miR-155 and miR-181a are stimulated and consequently activated by pro-inflammatory cytokines such as IL-1β and TNF-α [64] or downregulated by the application of a positive hydrostatic pressure [66]. In a similar manner, in this report, visfatin and resistin stimulus significantly increased the transcriptional levels of *miR-181a* and *miR-155* in OA chondrocyte cultures. In 2013, Sudedi et al. [67] demonstrated that murine RAW 264.7 macrophages incubated with recombinant human globular adiponectin significantly increased the expression levels of *miR-155* after 12 h of stimulus.

Mir-let7e play an important role in cellular proliferation, apoptosis, and inflammation by modulating the NF-κB signaling [68].

Serum levels of miR-let7 were found inversely correlated with the risk of joint arthroplasty, and this association was independent of age, sex, and BMI, leading to speculate miR-let7e as a potential biomarker for severe OA [69]. Furthermore, miR-let7e represents one of the most prominent miRNA implicated in metabolic syndrome and cardiovascular disorders [70,71].

In this study, we first showed a significant increase of *miR-let7e* gene expression in OA chondrocyte cultures stimulated with visfatin and resistin suggesting that miR-let7e contributes to explaining the high association between metabolic syndrome, cardiovascular disease, and OA [72,73].

The *NF-κB* signaling pathway plays a central role in inflammation and in cartilage degradation in OA [74]. In fact, the activated form of *NF-κB* induces the transcription of various catabolic factors, MMPs, ADAMTS, cytokines and chemokines, pro-inflammatory mediators, and contributes to the degradation of collagen and proteoglycans [74,75]. Different studies showed that visfatin and resistin induce the activation of the *NF-κB* signaling pathway [41,76,77,78,79]. In agreement with the current literature we demonstrated a significant increase of the expression, activation, and in turn of the nuclear translocation of the *NF-κB* p50 subunit in OA chondrocytes incubated by visfatin and resistin. Moreover, we found a direct interaction between *NF-κB* and visfatin and resistin stimulus after the inhibition of the *NF-κB* pathway by using a specific *NF-κB* inhibitor, BAY11-7082, as described by other authors [12,41,79]. Our data demonstrated that BAY11-7082, downregulating *NF-κB*, reduced the effect of the studied adipokines on *MMP-1*, *MMP-13*, and *Col2a1* expression. This finding indicates that visfatin, resistin, and BAY11-7082 are effective on chondrocyte metabolism through the regulation of the *NF-κB* signaling pathway.

In the present study, the effects of visfatin and resistin on the T/C-28a2 cell line were also investigated. We did not observe any significant modification in cell proliferation, apoptosis, as well as in the gene expression of the studied miRNA and *Col2a1* on the stimulated cells in comparison to baseline. Visfatin and resistin significantly increased *MMP-1* and *MMP-13* levels similarly to the results obtained by Guan et al. [80] on IL-1β-stimulated T/C-28a2; the adipokines also increased the gene expression of *miR-155* in T/C-28a2 in comparison to un-stimulated cells, while no results from the literature are reported to support this data.

Since there are difficulties and problems related to obtaining healthy human articular cartilage for culture experiments, various chondrocyte cell lines have been developed. Human T/C-28a2 is a line of immortalized cells isolated from human juvenile costal cartilage and is generated by a transfection procedure by using a retroviral vector pZipNeoSV (X) and the SV40 large T antigen. The immortalization of the cells allows the generation of stable lines that express and maintain the differentiated phenotype under defined conditions [81]. They are not the optimal substitution for primary articular chondrocytes, even if, their phenotypic characteristics are close to those of physiologic chondrocytes [82].

T/C-28a2 cells have been used in some studies to investigate chondrocyte-specific response patterns after stimulation with IL-1β or during loading experiments [83,84,85]. T/C-28a2-specific responses to cytokines, such as IL-1β or TNF-α, or adipokines, as resistin, were similar to those found in human normal chondrocyte cultures [12,81].

In our report, the lack of response of T/C-28a2 to visfatin and resistin stimulus is difficult to explain; one of the possible hypotheses could be a different expression of specific receptors for these adipokines in a cell line with respect to those in OA chondrocytes [36,86].

## 4. Materials and Methods

### 4.1. Chondrocyte Cultures

A human immortalized T/C-28a2 cell line was kindly provided by Goldring Laboratory and derived from human juvenile costal cartilage generated by transfection procedure using a retroviral vector pZipNeoSV (X) and the SV40 large T antigen as already described [80].

Human OA articular cartilage was obtained from femoral heads of five non-obese (BMI ranging from 20 to 23 Kg/m^2^) and non-diabetic patients (two men and three women, age ranging from 68 to 79) with hip OA defined by the clinical and radiological ACR criteria [87], undergoing surgery for total hip replacement. Osteoarthritis grades ranged from moderate to severe, and cartilage showed typical osteoarthritic changes, as the presence of chondrocyte clusters, fibrillation, and loss of metachromasia (Mankin degree 3–7) [88]. Osteoarthritis chondrocytes were derived from the area adjacent to the OA lesion. The femoral heads were supplied by the Orthopaedic Surgery, University of Siena, Italy. The Ethics Committee of the Azienda Ospedaliera Universitaria Senese/Siena University Hospital approved the use of human articular samples (decision no. 726/07), and all subjects signed an informed consent.

The chondrocytes were isolated immediately after surgery. In brief, cartilage fragments were aseptically dissected from each donor and processed by an enzymatic digestion with trypsin (Sigma–Aldrich, Milan, Italy) for 15 min at 37 °C and then with type IV collagenase (Sigma–Aldrich, Milan, Italy) for 12–16 h at 37 °C.

The obtained cell suspension was filtered twice using 70-μm nylon meshes, washed, and centrifuged for 5 min at 700× *g*. The viability was assessed by Trypan Blue (Sigma–Aldrich, Milan, Italy) test identifying 90% to 95% cell survival. Chondrocytes were recovered, seeded into 10-cm diameter tissue culture plates, and were expanded for 10–12 days in a monolayer incubator with 5% CO_2_ and 90% humidified atmosphere at 37 °C until it reached 80% confluence.

Cells were grown in Dulbecco’s Modified Eagle Medium (DMEM) (Euroclone, Milan, Italy), containing 10% Fetal Bovine Serum (FBS) (Euroclone, Milan, Italy), with 200 U/mL penicillin and 200 µg/mL streptomycin (P/S) (Sigma–Aldrich, Milan, Italy). The medium was changed every 3–4 days. The cell morphology was examined daily with an inverted microscope (Olympus IMT-2, Tokyo, Japan), and OA primary chondrocytes at the first passage were employed for the experiments to guarantee their phenotypic stability preserved which can occur when subcultured in monolayer [36]. For each single experiment a cell culture from a unique donor was used.

### 4.2. Treatment of Chondrocyte Cultures

T/C-28a2 cell line and human OA chondrocytes, at the first passage, were plated in 6-well dishes at a starting density of 6 × 10^6^ cells/well until they became confluent. Human recombinant visfatin (Sigma–Aldrich, Milan, Italy) and human recombinant resistin (BioVendor, Rome, Italy) were first dissolved in phosphate buffered saline (PBS) (Euroclone, Milan, Italy), according to the manufacturer’s instructions, and then they were diluted in the culture medium immediately before the treatment to reach the final concentration required.

The cells were treated for 24 h with visfatin at concentration of 5 μg/mL and 10 μg/mL or resistin 50 ng/mL and 100 ng/mL. The concentrations of the adipokines used in our in vitro study were selected according to those used by other authors [12,39]; the final concentrations were chosen based on the best results obtained in terms of viability.

After the treatment, the media were removed, centrifuged, and stored at −80 °C; the cells were immediately processed to carry out cell viability assay, flow cytometry analysis, and quantitative real-time PCR.

Afterwards, the cells were pre-incubated for 2 h with 1 μM BAY 11-7082 (*NF-κB* inhibitor, IKKα/β, Sigma–Aldrich, Milan, Italy) and then treated 24 h with the tested concentrations of visfatin and resistin. Subsequently, the gene expression of *MMP-1*, *MMP-13*, and *Col2a1* was evaluated.

### 4.3. MTT Assay

The viability of the cells was evaluated immediately after the treatment by MTT assay. Chondrocytes were incubated for 3 h at 37 °C in a culture medium containing 10% of 5 mg/mL MTT (3-[4,4-dimethylthiazol-2-yl]-2,5-diphenyl-tetrazoliumbromide) (Sigma–Aldrich, Milan, Italy). After the period of incubation, the medium was removed and 0.2 mL of dimethyl sulfoxide (DMSO) (Rottapharm Biotech, Monza, Italy) was added to each well to solubilize the formazan crystals. The absorbance was measured at 570 nm in a microplate reader (BioTek Instruments, Inc., Winooski, VT, USA). A control well without cells was employed for blank measurement.

The percentage of survival cells was evaluated:

% of survival cells = (absorbance of considered sample)/(absorbance of control) × 100

The experiments were performed on pre-confluent cell cultures in order to prevent contact inhibition which can condition the results. Data were reported as OD units per 10^4^ adherent cells.

### 4.4. Detection of Apoptosis

The evaluation of apoptotic cells was developed by using Annexin V-FITC and propidium iodide (PI) (ThermoFisher Scientific, Milan, Italy). T/C-28a2 cell line and OA chondrocyte were seeded in 12-well plates (8 × 10^4^ cells/well) for 24 h in DMEM with 10% FBS. Then, the medium was removed, and the cells were cultured in DMEM with 0.5% FBS usually used during the treatment procedure. After that, the chondrocytes were washed and harvested by using trypsin, collected into cytometry tubes, and centrifuged at 1500 rpm for 10 min. The supernatant was replaced, and the pellet was resuspended in 100 μL of 1 × Annexin-binding buffer, 5 μL of Alexa Fluor 488 annexin-V conjugated to fluorescein (green fluorescence) and 1 μL of 100 μg/mL propidium iodide working solution. They were added to 100 μL of cell suspension. Cells were incubated at room temperature for 15 min in the dark. Then, 600 μL of 1 × Annexin-binding buffer was added before the analysis at flow cytometer. A total of 10,000 events (1 × 10^4^ cells per assay) were measured by the instrument. The obtained results were analyzed with Cell Quest software (Version 4.0, Becton Dickinson, San Jose, CA, USA). The evaluation of apoptosis was carried out considering staining cells simultaneously with Alexa Fluor 488 annexin-V and propidium iodide; this allowed to discriminate intact cells (annexin-V and propidium iodide-negative), early apoptosis (annexin-V-positive and propidium iodide-negative), and late apoptosis (annexin-V and propidium iodide positives) [85].

The results were expressed as a percentage of positive cells to each dye (total apoptosis), and the data were represented as the mean of three independent experiments (mean ± standard deviation (SD).

### 4.5. RNA Isolation and Quantitative Real-Time PCR

Total RNA, including miRNA, was extracted using TriPure Isolation Reagent (Euroclone, Milan, Italy) according to the manufacturer’s instructions, and was stored at −80 °C. The concentration, purity, and integrity of RNA were evaluated by measuring the OD at 260 nm and the 260/280 and 260/230 ratios by Nanodrop-1000 (Celbio, Milan, Italy). The quality of RNA was verified by electrophoresis on agarose gel (FlashGel System, Lonza, Rockland, ME, USA). Reverse transcription for miRNA was carried out by the cDNA miScript PCR Reverse Transcription (Qiagen, Hilden, Germany), while the same procedure for target genes by QuantiTect Reverse Transcription Kit (Qiagen, Germany), according to the manufacturer’s instructions.

miRNA and target genes were examined by real-time PCR using, miScript SYBR Green (Qiagen, Hilden, Germany) and QuantiFast SYBR Green PCR (Qiagen, Hilden, Germany) kits, respectively. A list of the used primers is reported in Table 1. All qPCR reactions were achieved in glass capillaries by a LightCycler 1.0 (Roche Molecular Biochemicals, Mannheim, Germany) with LightCycler Software Version 3.5. The reaction procedure miRNA consisted 95 °C for 15 min for HotStart polymerase activation, followed by 40 cycles of 15 s at 95 °C for denaturation, 30 s at 55 °C for annealing, and 30 s at 70 °C for elongation, according to the protocol. Target gene amplification was performed at 5 in at 95 °C, 40 cycles of 15 s at 95 °C, and 30 s at 60 °C. In the final step of both protocols, the temperature was raised from 60 °C to 95 °C at 0.1 °C/step to plot the melting curve.

To further analyze the dissociation curves, we visualized the amplicons lengths in an agarose gel to confirm the correct amplification of the resulting PCR products.

For the data analysis, the *C*_t_ values of each sample and the efficiency of the primer set were calculated through LinReg Software [89] and then converted into relative quantities and normalized using the Pfaffl model [90].

The normalization was performed considering SNORD-25 for miRNA and HPRT-1 for target genes, as the housekeeping genes. These genes were chosen according to geNorm software version 3.5 [91].

### 4.6. Immunofluorescence

Osteoarthritis chondrocytes at the first passage were plated in coverslips in Petri dishes (35 × 10 mm) at a starting low density of 4 × 10^4^ cells/chamber, to prevent possible cell overlapping, and re-suspended in 2 mL of culture medium until 80% of confluence. The cells were processed after 2 h of treatment with adipokines to evaluate the potential activation of the *NF-κB* pathway. The chondrocytes were washed in phosphate-buffered saline (PBS) (Euroclone, Milan, Italy) and then fixed in methanol for 15 min and in acetone for 5 min at −20 °C. Afterwards, the cells were permeabilized with a blocking solution (PBS and 2% bovine serum albumin (BSA) (Sigma–Aldrich, Milan, Italy) for 20 min at room temperature, and then incubated overnight at 4 °C with mouse monoclonal anti-p50 subunit primary antibody (Santa Cruz Biotechnology, Italy) diluted at 1:50 in PBS and 0.1% BSA. Three washes in PBS of the coverslips were followed by 1 hr incubation with goat anti-mouse IgG-Texas Red conjugated antibody (Southern Biotechnology, Italy) diluted at 1:100 in PBS and 0.1% BSA. Finally, the coverslips were mounted with Vecta shield (Vector Labs). Fluorescence was examined under an AxioPlan (Zeiss, Oberkochen, Germany) light microscope equipped with epifluorescence at 200× and 400× magnification. The negative controls were obtained by omitting the primary antibody. Immunoreactivity of p50 was semi-quantified as the mean densitometric area of p50 signal into the nucleus and into the cytoplasm, by AxioVision 4.6 software measure program [92]. At least 100 OA chondrocytes from each group were evaluated.

### 4.7. Statistical Analysis

Three independent experiments were carried out and the results were expressed as the mean ± SD of triplicate values for each experiment. Data normal distribution was evaluated by Shapiro–Wilk, D’Agostino and Pearson, and Kolmogorov–Smirnov tests.

Data from real-time PCR were evaluated by one-way ANOVA with a Tukey’s post-hoc test using 2^−ΔΔCT^ values for each sample.

All analyses were performed through the SAS System (SAS Institute Inc., Cary, NC, USA) and GraphPad Prism 6.1. A significant value was defined with a *p*-value < 0.05.

## 5. Conclusions

Our data demonstrate for the first time the effect of visfatin and resistin in modulating the expression levels of some miRNA normally aberrantly expressed in OA. Moreover, the results show a significant decrease at transcriptional levels of the main proteases involved in OA pathogenesis inducing to speculate about the role of adipokines in cartilage degradation. The observed transcriptional modifications of miRNA and target genes seem to be due to the modulation of the NF-κB signaling pathway.

However, our report presents some limitations that have to be noticed. The results of the current study are limited to the lack of the same evaluation performed on normal primary chondrocytes. Moreover, additional experiments to better elucidate the direct relationship between visfatin, resistin, and the *NF-κB* signaling pathway modulation are required; for instance, transfection experiments with anti-miRNA could be useful in identifying the regulation of specific target genes, useful to elucidate their mechanism of action.

Taken together, these results confirm the complex relationship between adipokines and miRNA and contribute to explain the role of obesity in the pathogenesis of OA and the relevance of weight loss, confirming the importance of controlling body weight in the treatment of the disease.

## Figures and Tables

**Table 1:** Primers used for RT-qPCR.

**miRNA Gene**	**Cat. No. (Qiagen)**
*miR-34a*	MS00003318
*miR-140*	MS00003500
*miR-146a*	MS00003535
*miR-155*	MS00008778
*miR-181a*	MS00006692
*miR-let7e*	MS00031801
**Target Gene**	**Cat. No. (Qiagen)**
*MMP-1*	QT00014581
*MMP-13*	QT00001764
*Col2a1*	QT00049518
